# Exploring the Intensity and Continuity of Hospital Care for Patients With Long Covid: Evidence From an English Urban Healthcare System

**DOI:** 10.1111/hex.70527

**Published:** 2026-02-11

**Authors:** Jonathan Clarke, Sneha Jha, Denys Prociuk, Erik Mayer, Simon de Lusignan, Nikki Smith, Ruairidh Milne, Cassie Lee, Johannes De Kock, Manoj Sivan, Brendan C. Delaney, LOCOMOTION Study Consortium

**Affiliations:** ^1^ I‐X, Imperial College London London UK; ^2^ Department of Mathematics, Imperial College London Centre for Mathematics of Precision Healthcare London UK; ^3^ iCARE Secure Data Environment, Imperial College Healthcare NHS Trust & Faculty of Medicine Imperial College London UK; ^4^ Nuffield Department of Primary Care Health Sciences University of Oxford Oxford UK; ^5^ Person with Long Covid, Member of the LOCOMOTION Study Patient Advisory Group Windsor UK; ^6^ Person with Long Covid; and Emeritus Professor of Public Health University of Southampton Southampton UK; ^7^ Imperial College London NHS Trust, Imperial College Respiratory Research Unit (ICRRU) London UK; ^8^ NHS Highland, COVID Recovery Service Inverness UK; ^9^ Academic Department of Rehabilitation Medicine University of Leeds Leeds UK

## Abstract

**Background:**

Long Covid (LC) is a multisystem condition leading to a wide range of symptoms and often requiring treatment by several different clinical specialties. Patients with LC have reported difficulties in accessing care and a lack of coordination of their care, particularly in a hospital setting.

**Objective:**

To determine the extent to which the intensity and continuity of hospital care changes for patients after they receive an LC diagnosis.

**Design:**

Retrospective observational cohort study using a linked primary and secondary care dataset.

**Setting and Participants:**

Routine healthcare data from North West London Integrated Care System of patients with a recorded diagnosis of LC who had attended a secondary care hospital Trust from 1 January 2019 to 30 September 2023.

**Main Variables Studied:**

The intensity of utilisation of secondary care was calculated, and the continuity of care with respect to hospitals and specialties was computed using the sequential continuity score (SeCon) before the Covid‐19 pandemic, before and after an LC diagnosis.

**Results:**

5611 out of 6270 (90.1%) patients diagnosed with LC had a recorded secondary care interaction in the study period. Intensity of secondary care utilisation increased markedly in outpatient, inpatient and Emergency Department pathways after a diagnosis of LC but peaked in the week of diagnosis. Average hospital SeCon fell significantly after an LC diagnosis from 1.00 to 0.83, while specialty SeCon remained unchanged from after diagnosis (0.40) and before the pandemic (0.44). A notable shift in specialty activity was observed with a focus on respiratory medicine as a major hub in a densely connected patient‐sharing network with cardiology and other medical and surgical specialties.

**Discussion:**

A recorded LC diagnosis was associated with increases in the intensity of hospital activity and a reduction in hospital‐level care continuity, but no change in specialty continuity, which remains low.

**Conclusion:**

Collectively, this indicates a significant need to support LC patients as they navigate fragmented secondary care pathways.

**Patient and Public Contribution:**

This study was co‐designed with, conducted with and written in conjunction with people with long Covid.

## 1 Introduction

Long Covid (LC) is a multisystem condition, first identified after the onset of the Covid‐19 pandemic in patients whose symptoms, attributable to Covid‐19, persisted for many weeks and months after their initial acute infection [1, 2]. LC may lead to a wide range of symptoms and clinical signs, including tachycardia, fatigue, perceived cognitive impairment and joint pains, and affects between 3% and 6% of the UK population [3, 4, 5]. Each of these clinical features may require investigation and management by a separate medical specialty, and as such, coordination of care may be particularly challenging for patients with, or suspected of having, LC.

LC clinics were established in locations across the NHS in England and internationally to assist in the coordination of investigations and treatments for patients suspected of having LC and to refer patients to other specialties as required [6]. As this patient group's symptoms often include not only short‐term (4–12 weeks) but also longer‐term (beyond 12 weeks) symptoms that can interact with other long‐term conditions, the concept of continuity of care, often defined as quality of care over time, becomes of interest not only to patients, but also to service providers and policymakers [7].

Continuity of care describes the extent to which a patient attends the same service or clinical location or is seen by the same clinician over time [8, 9, 10]. In primary care, continuity of care is associated with better clinical outcomes and improved patient experience, while in secondary care, the relationship between continuity of care and patient outcomes is still being established [9, 11]. Despite this, patients, particularly those with multiple long‐term conditions (MLTCs), are concerned about fragmentation of care leading to clinical errors and a lack of oversight on connected problems that span several clinical specialties [12]. The Academy of Medical Sciences voiced particular concern regarding continuity of care for patients with MLTC who often attend several different providers to meet their healthcare needs [13]. Despite strong anecdotal evidence from our study's Patient Advisory Group and from recent qualitative studies of an increased secondary care burden and a decrease in care continuity, these changes have not been quantified empirically [14, 15].

### 1.1 Aims

This study aimed to investigate how patients who received a diagnosis of LC used secondary care services. It firstly aimed to examine the changing intensity of utilisation of outpatient, inpatient and ED services in the period before the Covid‐19 pandemic, and before and after an LC diagnosis was made. Thereafter, it explored the extent to which the clinical specialties attended changed over these three periods. Understanding the utilisation of secondary care services and the coordination of patient care can help us to shed light on the epidemiology of LC, as well as the help‐seeking behaviour of patients with LC and the clinical specialties in which LC patients are seen most frequently. This study is looking to improve patient care, by providing an indication of where potential future changes in healthcare provision could lie to improve efficiency to benefit both patients and healthcare providers. This study is a part of the LOng COvid Multidisciplinary consortium Optimising Treatments and servIces acrOss the NHS (LOCOMOTION) consortium, which aims to optimise assessment and treatment across LC services in the United Kingdom [16].

## 2 Methods

This is a retrospective observational study using linked health records data from primary and secondary care.

### 2.1 Data Sources

The Whole Systems Integrated Care (WSIC) database, hosted in the iCARE Secure Data Environment, was the primary source of data used for this study. The WSIC database contains record‐level data derived from primary care records of over 2.8 million patients registered to 350 GP practices in North West London and linked to hospital Secondary Uses Service data containing records of appointments, Emergency Department (ED) attendances and inpatient admissions. Only the first episode of inpatient admissions was included, and only outpatient appointments where the patient was seen were included. Records of attendance at LC clinics were separately provided by the North West London Integrated Care System. This dataset contains the dates of attendance and site identifier for appointments at any LC clinic in North West London. All datasets were linked to one another in the WSIC secure environment using a unique patient identifier.

### 2.2 Data Analysis

Patients were identified as having LC if their primary care record contained one of four LC UK SNOMED clinical terms disorder or diagnosis codes: 1325161000000102 (Post‐Covid‐19 syndrome), 1325181000000106 (Ongoing symptomatic Covid‐19), 1119303003 (Post‐acute Covid‐19) and 1119304009 (Chronic post‐Covid‐19 syndrome) on or after 1 January 2020. These codes were chosen based on their suggested use in clinical coding by NHS England upon their creation as SNOMED codes in January 2021, as used elsewhere [17].

To explore changes in clinical activity over time, we defined three distinct time periods, namely ‘pre‐pandemic’, ‘pre‐diagnosis’ and ‘post‐diagnosis’. The ‘pre‐pandemic’ period spans the year from 1 January 2019 to the 31 December 2019. The ‘pre‐diagnosis’ period is from 1 January 2020 up to the date an LC diagnosis code first appears in a patient's primary care record, and the ‘post‐diagnosis’ period is from the date an LC diagnosis first appears in a patient's record to the end of the study period on 30 September 2023.

### 2.3 Intensity of Care

First, we quantified the number of inpatient admissions, outpatient appointments and ED attendances per week for the period from 1 January 2019 to 30 September 2023. Then, we quantify the intensity of secondary care activity across each of these three pathways in relation to the date an LC diagnosis first appears in a patient's primary care record. This measure is calculated as the total clinical activity in a given pathway in each week in the year before and after an LC diagnosis first appears in a patient's record, divided by the number of patients with that duration of data available. To aid interpretation, values are transformed from events per patient per week to events per thousand patients per day.

### 2.4 Continuity of Care

We then focused on the continuity of outpatient clinical care in more detail. Outpatient appointments account for around 75% of all secondary care activity, and based on discussion with the Patient Advisory Group for the project, are a particular source of variation in the experiences of patients with LC. We began analysis of the outpatient clinic appointments through describing the number and proportion of appointments to each of the 30 most commonly attended clinical specialties in the pre‐pandemic, pre‐diagnosis and post‐diagnosis period.

We then consider outpatient continuity of care separately with respect to the provider of care and the clinical specialty providing care. The specialty providing care was defined by the Treatment Specialty code recorded against the appointment in the outpatient SUS record, and the provider is defined by the 3‐character hospital trust provider code.

We examined continuity of care using two methods, firstly the number of providers or specialties attended and secondly the Sequential Continuity index, or SeCon [10]. While the number of providers or specialties attended by a patient in a given period gives a measure of the overall spread of a patient's activity, it is unable to account for the temporal ordering of events. As an example, Patient A attending five appointments with Respiratory Medicine followed by five appointments with Cardiology may experience higher continuity of care than Patient B who alternated between Respiratory and Cardiology appointments five times, despite the number of specialties attended being the same.

Patient A: RRRRRCCCCC

Patient B: RCRCRCRCRC

To address this, SeCon is calculated as the number of times a patient attended the same specialty or provider on adjacent appointments, divided by the total number of appointments minus 1:

(1) $$\mathrm{SeCon}=\;\frac{\sum_{i=1}^{n-1}c_{i}}{n-1}$$

where *n* is the total number of visits and *c*_*i*_ is equal to 1 if visits *i* and *i* + *1* are to the same provider or specialty and 0 otherwise. In the above dummy example, the first patient would thus have a SeCon score of 8/(10−1) = 0.89, while the second patient would have a SeCon score of 0/(10−1) = 0.

The distribution of continuity of care measures between pre‐pandemic, pre‐diagnosis and post‐diagnosis periods was displayed using letter‐value plots (a variant of box plots providing further detail on the tails of distributions) [18]. Statistical differences between time periods were determined using two‐sided Mann–Whitney *U* tests. We further examine differences in these continuity measures for those patients who have a recorded attendance at an LC clinic, as defined by the presence of an outpatient clinic attendance in the LC clinic dataset.

### 2.5 Inter‐Specialty Continuity Networks

In the above example of Patient B, their alternating between Respiratory Medicine and Cardiology appointments may lead to fragmentation of clinical information or clinical responsibility for a patient between these two specialties. To identify across our data which specialties are related to each other in this way, we calculate the number of times patients attend each pair of specialties consecutively in the pre‐pandemic, pre‐diagnosis and post‐diagnosis periods between the 30 most commonly attended specialties across the study period. This produces a weighted, undirected network of 30 nodes, connected by edges corresponding to the number of times patients consecutively attended this pair of specialties, in either order. This network is constructed for the pre‐pandemic, pre‐diagnosis and post‐diagnosis periods and visualised in circular network plots.

### 2.6 Statistical Methods

Continuous variables were described using the median and 25th and 75th centiles and represented through a combination of tables, line charts, bar charts and letter value plots. Where appropriate, count variables were also represented as proportions. Differences in continuity of outpatient clinic care in the post‐diagnosis period according to age, gender, ethnicity and area‐level Index of Multiple Deprivation quintile were examined using the Kruskal–Wallis test.

### 2.7 Software

Data extraction was performed using Microsoft SQL Server Management Studio 2018, and data processing and analysis were conducted using Python version 3.7.9 and the Pandas version 1.3.2 and numpy version 1.19.5 libraries. Statistical tests were conducted using scipy version 1.5.4, and networks were constructed and visualised using NetworkX version 2.6.3.

## 3 Results

A total of 6170 patients were recorded as having a diagnosis of LC in the period from 1 January 2020 to 30 September 2023, from a total of 1,838,363 adult patients registered to primary care practices within the WSIC dataset on 1 January 2020. Of these, 5561 (90.1%) had contact with secondary care services between 1 January 2019 and 30 September 2023 with a total of 117,671 attendances recorded in total, equating to an average of 2.11 interactions per patient. Of these, 88,270 (75.0%) were outpatient appointments, 11,960 (10.2%) were inpatient admissions and 17,441 (14.8%) were ED presentations. Table S1 shows the demographic characteristics of these patients. See Prociuk et al. (2025) for details on the characteristics of the overall NW London population and factors predictive of an LC diagnosis [17].

### 3.1 Intensity of Secondary Care Activity

**Figure 1:**
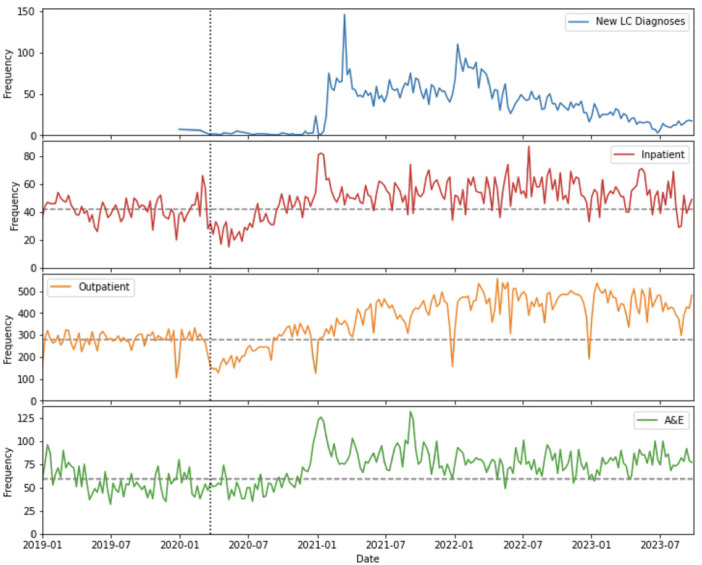
Frequency of new long Covid diagnoses and total activity for long Covid patients for each pathway in each week from 1 January 2019 to 30 September 2023.

The number of patients diagnosed with LC increases rapidly with the introduction of LC SNOMED disorder clinical terms codes in early 2021, with a further peak in early 2022, followed by a gradual decline in new diagnoses over the remainder of the study period (Figure 1). Some diagnoses were recorded with dates in 2020, before the introduction of relevant clinical codes. These cases were also included to reflect a likely small number of backdated diagnoses.

**Figure 2:**
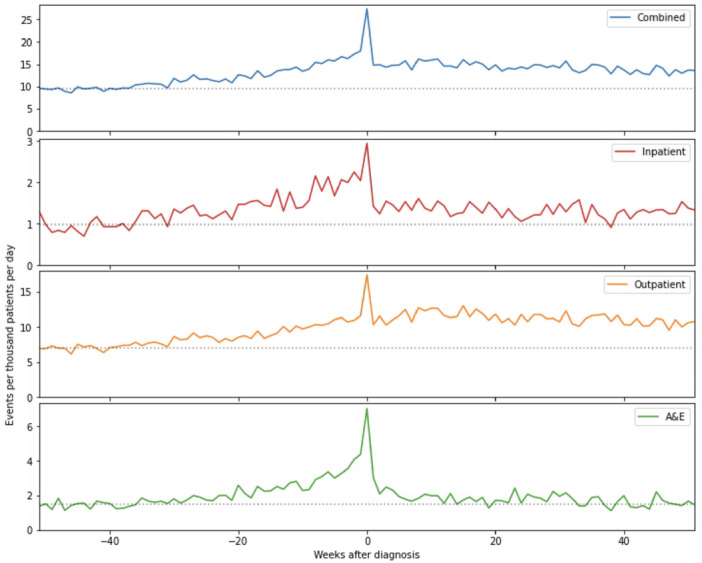
The number of recorded events for each pathway for each week in the year before and after the date of long Covid diagnosis in the primary care record. The dashed horizontal line indicated the average activity in weeks −52 to −40, the period 12 months to 9 months before a long Covid diagnosis was made.

As shown in Figure 2, the period of greatest secondary care activity occurs in the week a patient's LC diagnosis is recorded in their primary care record, with clear peaks seen across all secondary care pathways. In the pre‐diagnosis period, an increase in activity in all pathways up to a peak in the week of diagnosis is observed. After diagnosis, the intensity of activity falls to the early pre‐diagnosis baseline in the case of ED attendances, to a new baseline above early pre‐diagnosis intensity for inpatient admissions and to a new baseline above the late pre‐diagnosis intensity for outpatient appointments.

**Figure 3:**
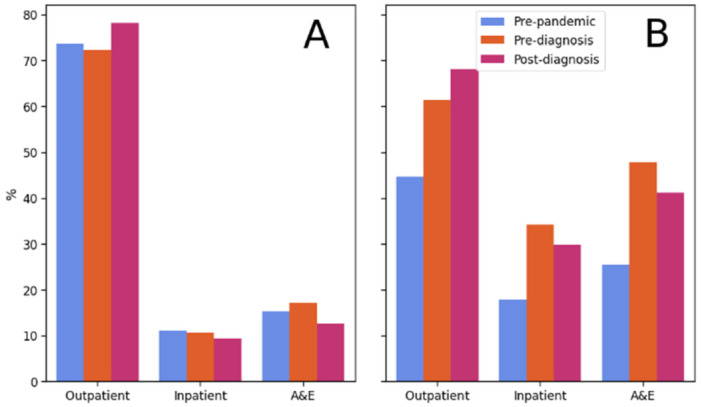
Panel A shows the percentage of activity in each time period in outpatient, inpatient and ED services (i.e., all ‘pre‐pandemic’ bars sum to 100%). Panel B shows the percentage of patients with recorded outpatient, inpatient and ED activity in each period.

The percentage of activity accounted for by outpatient appointments increases from 73.6% in the pre‐pandemic period to 78.1% in the post‐diagnosis period (Figure 3). Correspondingly, inpatient and ED activity fell from 11.1% to 9.4% and 15.3% to 12.5%, respectively. The share of ED activity is highest during the pre‐diagnosis period, where it accounts for 17.0% of all activity.

Over half of all patients (*n* = 3282, 53.2%) had a recorded secondary care attendance in the pre‐pandemic period, compared to 4452 (72.2%) in the pre‐diagnosis period and 4613 (74.8%) in the post‐diagnosis period. Further, the percentage of patients who had an outpatient appointment increased from 44.7% in the pre‐pandemic period to 61.4% in the pre‐diagnosis period and 68.0% in the post‐diagnosis period. The proportions of patients with a recorded inpatient admission (34.1%) or ED attendance (47.8%) were highest in the pre‐diagnosis period.

As shown in Table 1, the proportion of outpatient activity accounted for by each specialty varied between the pre‐pandemic, pre‐diagnosis and post‐diagnosis periods. Notable changes in activity between these periods include an increase in Respiratory Medicine activity from 4.1% of all outpatient appointments in the pre‐pandemic period to 10.9% in the pre‐diagnosis period and 14.6% in the post‐diagnosis period. Substantial increases in activity were also observed for Cardiology (6.0%, 7.8% and 8.9%) and Adult Mental Illness (0.6%, 2.8% and 3.4%). Decreases in activity are noted for most surgical specialties and Obstetrics and Gynaecology.

**Table 1:** Count of attendances to the 30 most frequently attended clinical specialties for outpatient care in the pre‐pandemic, pre‐diagnosis and post‐diagnosis periods. NOC—Not Otherwise Coded.

Specialty	Pre‐pandemic		Pre‐diagnosis		Post‐diagnosis	
	*N*	%	*N*	%	*N*	%
Respiratory Medicine	547	4.07	3540	10.85	5480	14.62
Allied Health Professional	1121	8.34	2737	8.39	3680	9.82
Cardiology	811	6.03	2539	7.78	3344	8.92
General Surgery	1111	8.26	2100	6.44	2395	6.39
Trauma & Orthopaedics	1015	7.55	1697	5.20	1880	5.01
Ophthalmology	947	7.04	1794	5.50	1665	4.44
Gynaecology	889	6.61	1492	4.57	1585	4.23
Gastroenterology	552	4.10	1348	4.13	1412	3.77
Radiology	566	4.21	1080	3.31	1410	3.76
Dermatology	664	4.94	1134	3.48	1114	2.97
Midwife Episode NOC	574	4.27	1144	3.51	943	2.52
ENT	493	3.67	851	2.61	1240	3.31
Nursing Episode NOC	471	3.50	962	2.95	939	2.50
General Medicine	243	1.81	1067	3.27	1050	2.80
Anaesthetics (pain)	253	1.88	926	2.84	1094	2.92
Adult Mental Illness	76	0.57	920	2.82	1267	3.38
Rheumatology	443	3.29	929	2.85	843	2.25
Clinical Haematology	308	2.29	947	2.90	865	2.31
Urology	355	2.64	823	2.52	765	2.04
Endocrinology	381	2.83	729	2.23	743	1.98
Neurology	373	2.77	762	2.34	693	1.85
Nephrology	232	1.73	555	1.70	492	1.31
Obstetrics	334	2.48	505	1.55	430	1.15
Medical Oncology	138	1.03	500	1.53	478	1.27
Plastic Surgery	186	1.38	314	0.96	249	0.66
Acute Internal Medicine	25	0.19	225	0.69	450	1.20
Haematology	28	0.21	365	1.12	304	0.81
Medical Ophthalmology	123	0.91	218	0.67	244	0.65
Audiological Medicine	108	0.80	212	0.65	220	0.59
Accident & Emergency	81	0.60	205	0.63	217	0.58
Total	13,448		32,620		37,491	

### 3.2 Continuity of Secondary Care

Out of a total of 11,677 pairs of consecutive outpatient appointments in the pre‐pandemic period, 8513 (72.9%) were between the same hospital trust and 4806 (41.2%) were between the same specialty. In the pre‐diagnosis period, out of 30,572 consecutive appointments, 22,357 (73.1%) were between the same hospital trust and 12,567 (41.1%) between the same specialty. In the post‐diagnosis period, of 35,239 consecutive appointments, 24,976 (70.9%) were between the same trust and 13,795 (39.2%) were between the same specialty.

**Figure 4:**
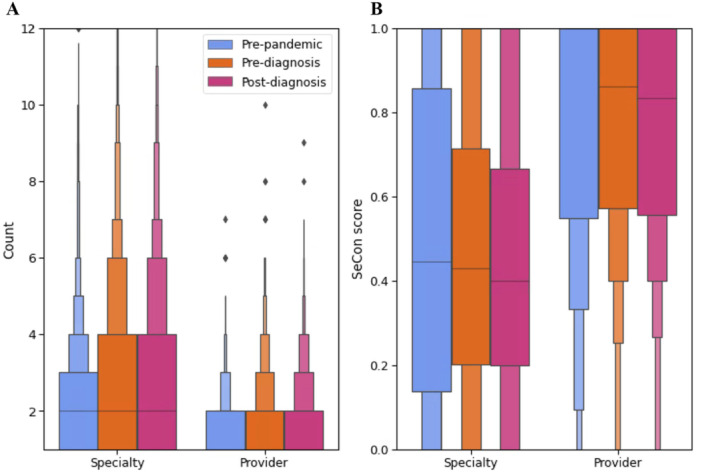
Panel A shows letter‐value plots for the number of clinical specialties attended and the number of hospital trusts attended in the pre‐pandemic, pre‐diagnosis and post‐diagnosis periods for outpatient care. Panel B shows letter‐value plots for the SeCon score for clinical specialties and hospital trusts in the pre‐pandemic, pre‐diagnosis and post‐diagnosis periods for outpatient care.

Compared to before the Covid‐19 pandemic (median = 2, IQR: 1–3), of those who received outpatient care, the number of specialties attended was higher in the pre‐diagnosis (median = 2, IQR: 1–4, *p* < 0.001) and post‐diagnosis (median = 2, IQR: 1–4, *p* < 0.001) periods (Figure 4A). There was no statistically significant difference in SeCon scores when comparing the pre‐pandemic period (median = 0.44, IQR: 0.14–0.86) to the pre‐diagnosis (median = 0.43, IQR: 0.20–0.71, *p* = 0.623) and post‐diagnosis periods (median = 0.40, 0.20–0.67, *p* = 0.204) (Figure 4B).

Of those receiving outpatient care, the number of hospital trusts attended also increased significantly from the pre‐pandemic to the pre‐diagnosis and post diagnosis periods (*p* < 0.001 in both cases). The percentage of patients attending three or more trusts increased from 10.2% in the pre‐pandemic period to 18.9% in the pre‐diagnosis period and 19.0% in the post‐diagnosis period (Figure 4A). SeCon scores were significantly lower in the pre‐diagnosis (median = 0.86, IQR: 0.57–1.00, *p* = 0.003) and post‐diagnosis (median = 0.83, IQR: 0.56–1.00, *p* < 0.001) periods than the pre‐pandemic period (median = 1.00, IQR: 0.55–1.00) (Figure 4B).

In the post‐diagnosis period, age was significantly associated with differences in the number of attendances and continuity of care, with frequency of appointments increasing and continuity of care decreasing with increasing age (all *p* < 0.001, Table S2). Differences in continuity of care were not observed according to ethnicity, while statistically significant differences (*p* = 0.020) were observed according to gender in the count of specialties attended (women attending more specialties than men). Significant differences (*p* = 0.029) were also observed according to area‐level socio‐economic deprivation in specialty SeCon where an inconsistent pattern is observed across quintiles of deprivation (Table S2).

A total of 1114 patients (18.1%) had a recorded attendance at an LC clinic. As shown in Table 2, these patients attended more clinical specialties and more providers than those who did not attend an LC clinic. Patients who had a recorded attendance at an LC clinic had significantly lower care continuity and attended significantly more specialties and providers (*p* < 0.001 in all cases) than those who did not.

**Table 2:** Summary statistics comparing continuity of care measures for patients in the pre‐pandemic, pre‐diagnosis and post‐diagnosis periods and with and without a recorded attendance at a long Covid clinic in the post‐diagnosis period.

	Pre‐pandemic	Pre‐diagnosis	Post‐diagnosis	Attended a long Covid clinic	
				Yes	No
Patients	2759 (44.7%)	3786 (61.4%)	4197 (68.0%)	1114 (18.1%)	5056 (81.9%)
Outpatient attendances	3 (2–7)	5 (2–11)	5 (2–12)	6 (3–13)	5 (2–12)
Number of specialties attended	2 (1–3)	2 (1–4)	2 (1–4)	3 (2–5)	2 (1–4)
Number of providers attended	1 (1–2)	1 (1–2)	1 (1–2)	2 (1–3)	1 (1–2)
Specialty SeCon	0.44 (0.14–0.86)	0.43 (0.20–0.71)	0.40 (0.20–0.67)	0.38 (0.20–0.60)	0.40 (0.20–0.73)
Provider SeCon	1.00 (0.55–1.00)	0.86 (0.57–1.00)	0.83 (0.56–1.00)	0.75 (0.50–1.00)	0.88 (0.57–1.00)

**Figure 5:**
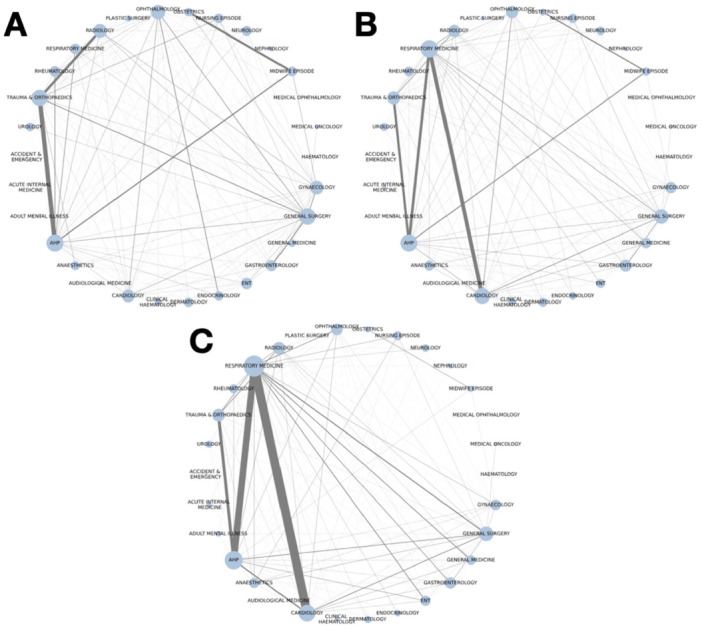
Visualisations of the outpatient speciality continuity network in the pre‐pandemic (A), pre‐diagnosis (B) and post‐diagnosis (C) periods. Nodes correspond to each of the 30 most commonly attended outpatient specialties. Node size is proportional to its weighted degree. Edge weights correspond to the percentage of all discontinuous pairs of attendances in each network.

In the pre‐pandemic period, where patients consecutively attended different outpatient clinics, this was most likely to be between trauma and orthopaedics and allied health professionals (AHPs) (193 events, 3.3%), trauma and orthopaedics and radiology (146 events, 2.5%), and obstetrics and midwife appointments (134 events, 2.3%). In the pre‐diagnosis period, connections between respiratory medicine and cardiology (491 events, 3.1%), respiratory medicine and AHPs (415 events, 2.6%), and trauma and orthopaedics and AHPs (390 events, 2.4%) are most common. In the post‐diagnosis period, respiratory medicine is more strongly connected to cardiology (890 events, 4.6%) and AHPs (772 events, 4.0%). In all three periods, interactions between a wide range of clinical specialties occur, with 82.5% of all possible pairs of providers connected in the pre‐pandemic period, 94.7% in the pre‐diagnosis period and 95.6% in the post‐diagnosis period (Figure 5).

## 4 Discussion

Our study investigated the utilisation of secondary care services by patients who received a diagnosis of LC. Our findings indicate that receipt of an LC diagnosis is associated with a shift in the way a patient interacts with secondary care services. Across inpatient, ED and outpatient services, receipt of an LC diagnosis is associated with an increase in activity compared to before the Covid‐19 pandemic.

Notably, the weeks leading up to an LC diagnosis are associated with a substantial increase in the intensity of secondary care activity, peaking in the week that an LC diagnosis is recorded. This finding suggests that secondary care is likely to be an important means by which an LC diagnosis is received, not only through outpatient clinics, but also via ED attendances and inpatient admissions, in turn filtering through to primary care records.

Most secondary care activity is through outpatient appointments, and this remains stable across the pre‐pandemic, pre‐diagnosis and post‐diagnosis periods. However, our findings reveal that of those patients receiving outpatient care, the number of hospital trusts and specialties attended significantly increases from the pre‐pandemic to the post‐diagnosis periods, likely reflecting the multisystem nature of LC. This finding is associated with a decrease in sequential continuity of care. Similar experiences of discontinuity following diagnosis are reported in patients with diabetes and those with MLTCs [12, 19]. Increasing age was consistently associated with lower continuity of care, while clear trends in continuity of care according to ethnicity, gender and socio‐economic deprivation were not observed.

Adding to this, those patients with a recorded attendance at an LC clinic also had more apparently fragmented outpatient care than those who did not. This finding can be explained in several ways. Firstly, in the absence of being able to account for differences in clinical need, it is not possible to discern the impact of LC clinics on continuity of care, specifically as LC clinics are generally provided by existing clinical services, such as respiratory medicine, without specific LC clinic flags in the medical record. Secondly, many LC clinics may be delivered through community or primary care settings and may not be included in the LC clinic dataset provided. This may also explain the low proportion (18.1%) of LC patients with a recorded LC clinic attendance.

Further, this finding might reflect unmet patient needs, in keeping with reports from people with LC describing difficulties in accessing healthcare and an insufficient response to and recognition of their illness when they do access LC clinics [20]. Finally, in the case of LC clinics, apparently more fragmented care may result from an increase in referral to other specialities, aligned with these clinics' goal of coordinating services for those diagnosed with LC [6]. This decrease in continuity may reflect necessary input from a range of clinical specialists and may be beneficial to patients. However, its potential impact on patients to navigate often convoluted journeys between specialties and hospital trusts to obtain this expertise should be considered by primary and secondary care providers.

The specialties between which patients are shared change markedly from the pre‐pandemic to the post‐diagnosis periods, with a relative shift from maternity and orthopaedic services to respiratory medicine and cardiology services. Whilst this finding can partially be explained by the commonly reported persistent physical LC symptoms being related to heart and circulatory problems and breathing difficulties [3], patients' healthcare usage behaviour also provides us with glimpses into living with LC. These changes in specialities may indicate a transition from living a normal, otherwise healthy life to living with one or more long‐term conditions.

Our findings indicate that the continuity of care between hospital specialties has not changed dramatically from before the pandemic to after an LC diagnosis, remaining common across this time. Although specialty continuity does not decrease after a diagnosis of LC, it is likely that for many patients the ability to effectively navigate similarly fragmented outpatient care may become more difficult after their LC diagnosis.

### 4.1 Strengths and Limitations

A key strength of this study is its focus on using administrative healthcare data to quantify the intensity and continuity of secondary care for LC patients. This is a markedly understudied area of research that is of importance to LC patients and also other patients with long‐term conditions [12, 20]. This provides a valuable quantitative corroboration for many of the experiences of patients with LC, including those from the Patient Advisory Group of the LOCOMOTION study.

As described in Prociuk et al. [17], the patients included in this study are only those who have a recorded diagnosis of LC in their primary care record. At around 0.3% of adults in the dataset, this represents a significant under‐coding compared to the estimated 3%–10% of the UK adult population living with persistent symptoms following Covid‐19 infection [4, 5, 21]. The study is limited to patients living in North West London, part of a densely populated conurbation with many nearby hospitals. This may particularly lead to lower provider continuity than in areas with a single hospital trust nearby, and our findings may not represent continuity of care across the country [22, 23].

As a retrospective observational study of administrative healthcare data, this study is unable to determine to what extent continuity of care is perceived as beneficial or problematic for LC patients. While extensive evidence supports improved patient experience from continuity of care, there is also evidence to suggest that a breadth of clinical opinions, often provided by other specialties or hospitals, may be associated with improved clinical outcomes [9, 24, 25]. This is particularly the case with LC, given the different organ systems being affected in the same individual.

In the case of LC, where patients have reported challenges in having their symptoms recognised by some clinicians, being referred to a clinician who takes their condition seriously may be more than worth the fragmentation of care it produces [20]. Further work is required to understand how LC patients perceive the burdens and opportunities of fragmentation of their care. The low proportion of patients with a recorded LC clinic attendance in the study (18.1%) is of particular concern, given NHS England's investment in 90 specialist clinics to cover the entire population of England. This low percentage may be the result of genuinely low rates of referral to and attendance at LC clinics, or may be the result of incomplete data on clinic attendance for a service spanning primary, secondary and community care settings.

### 4.2 Implications for Policy and Practice

Patients with LC, as indeed other LTCs, frequently see different clinical specialties at different hospitals, especially in areas like NW London with specialties often split across different local hospitals, contributing to fragmented clinical care. This may place a significant organisational and physical, cognitive and emotional burden on patients and their carers who may already be struggling with managing the symptoms associated with LC. Services should pay particular attention to ensure integration and coordination of care for patients with LC across healthcare tiers so that they are supported to attend the range of appointments they are referred to and that their clinical information is available to all clinicians they see [26].

Educating clinicians in primary and secondary care about the potential increased burden of care activity and challenges of care coordination patients with LC may experience is important and is also applicable to other long‐term conditions. There may be a particular opportunity here for the effective use of care coordinators in primary care to assist patients and clinicians. How the personal burden of attending clinical appointments is felt differently across patients with LC and other long‐term conditions is an important area for future work. Similarly, understanding how patients with LC may experience positive and negative effects of continuity of care across the course of their illness may provide an important opportunity to prioritise continuity of care at critical points in a patient's trajectory and less so at other times.

The finding of LC diagnoses coinciding with high secondary care activity in acute pathways, in addition to outpatient appointments, poses the unanswered question of how LC diagnoses are made and, in turn, recorded in a patient's medical record. The peak in ED attendances in the week of an LC diagnosis may indicate patients presenting to ED, where an LC diagnosis is made or referral from primary care to ED after an LC diagnosis is made in the context of urgent clinical need. Despite using linked primary and secondary care records, this question of cause or effect is not clearly answerable and may instead require a qualitative approach.

The patients included in this study likely represent a small and potentially biased fraction of those with LC due to incomplete coding of LC diagnoses in primary care. Efforts should continue to improve the recording of LC diagnoses in medical records, irrespective of the current status of a patient's symptoms. Providers should also consider opportunities for patients to contribute this information to their own medical records to reduce the current under‐recording of this condition. As LC remains a relatively new condition, ongoing research is needed to understand how patterns of secondary care utilisation for LC patients change over time, particularly regarding the intersection between LC and other long‐term conditions.

## 5 Conclusion

Receiving a diagnosis of LC contributes to an increase in the intensity of interactions with secondary care providers. In turn, this leads to a significant increase in the number of different specialists a patient sees and a reduction in their hospital‐level continuity of care. While continuity of care with respect to clinical specialties is low before the Covid‐19 pandemic, it remains low after an LC diagnosis, when a patient's need for improved continuity may increase. Efforts to better understand the burdens and opportunities of fragmentation of hospital care for LC patients and the development of integrated care pathways are crucial in ensuring patients are supported as they navigate often convoluted clinical trajectories.

## LOCOMOTION Study Consortium

Nawar Bakerly: Principal Investigator; Kumaran Balasundaram: NHS Clinical Research Fellow; Megan Ball: NHS Clinical Research Fellow; Mauricio Barahona: Co‐Investigator; Alexander Casson: Co‐Investigator; Jonathan Clarke: HEI Researcher; Karen Cook: Patient Advisory Group Member; Rowena Cooper: NHS Clinical Research Fellow; Vasa Curcin: Co‐Investigator; Julie Darbyshire: Co‐Investigator; Helen Davies: Principal Investigator; Helen Dawes: Co‐Investigator; Simonde Lusignan: Co‐Investigator; Brendan Delaney: Chief Investigator; Carlos Echevarria: Principal Investigator; Sarah Elkin: Principal Investigator; Ana Belen Espinosa Gonzalez: HEI Researcher; Rachael Evans: Principal Investigator; Sophie Evans: Patient Advisory Group Member; Zacchaeus Falope: Principal Investigator; Ben Glampson: HEI Researcher; Madeline Goodwin: Research Assistant; Trish Greenhalgh: Co‐Investigator; Darren C. Greenwood: Co‐Investigator; Stephen Halpin: Principal Investigator; Juliet Harris; NHS Research Assistant; Will Hinton: HEI Researcher; Mike Horton: Co‐Investigator; Samantha Jones: NHS Clinical Research Fellow; Joseph Kwon: HEI Researcher; Cassie Lee: NHS Clinical Research Fellow; Ashliegh Lovett: NHS Clinical Research Fellow; Mae Mansoubi: HEI Researcher; Victoria Masey: NHS Clinical Research Fellow; Harsha Master: Principal Investigator; Erik Mayer: HEI Researcher; Bernardo Meza‐Torres: HEI Researcher; Ruairidh Milne: Patient Advisory Group Member; Ghazala Mir: Co‐Investigator; Jacqui Morris: Principal Investigator; Adam Mosley: NHS Research Assistant; Jordan Mullard: HEI Researcher; Daryl O'Connor: Co‐Investigator; Rory O'Connor: Co‐Investigator; Thomas Osborne: Project Manager; Amy Parkin: NHS Clinical Research Fellow; Stavros Petrou: Co‐Investigator; Anton Pick: Principal Investigator; Denys Prociuk: HEI Researcher; Clare Rayner: Patient Advisory Group Member; Amy Rebane: Patient and Public Involvement Manager; Natalie Rogers: Patient Advisory Group Member; Janet Scott: Principal Investigator; Manoj Sivan: Chief Investigator; Nikki Smith: Patient Advisory Group Member; Adam Smith: Statistician; Emma Tucker: Principal Investigator; Ian Tucker‐Bell: Patient Advisory Group Member; Paul Williams: NHS Clinical Research Fellow; Darren Winch: Patient Advisory Group Member; Conor Wood: NHS Research Assistant.

## Author Contributions

**Jonathan Clarke:** conceptualization, data curation, formal analysis, writing – original draft, writing – review and editing. **Sneha Jha:** conceptualization, data curation, formal analysis, writing – original draft, writing – review and editing. **Denys Prociuk:** conceptualization, data curation, formal analysis, writing – original draft, writing – review and editing. **Erik Mayer:** conceptualization, writing – review and editing. **Simon de Lusignan:** conceptualization, writing – review and editing. **Nikki Smith:** conceptualization, writing – review and editing. **Ruairidh Milne:** conceptualization, writing – review and editing. **Cassie Lee:** conceptualization, writing – review and editing. **Johannes De Kock:** conceptualization, writing – original draft, writing – review and editing. **Manoj Sivan:** conceptualization, writing – review and editing. **Brendan C. Delaney:** conceptualization, supervision, writing – review and editing.

## Disclosure

The views expressed in this publication are those of the authors and not necessarily those of NIHR, the Department of Health and Social Care, or NHS England.

## Ethics Statement

Ethical approval for this study was granted by the Yorkshire & The Humber—Bradford Leeds Research Ethics Committee (reference: 21/YH/0276) and the Health Research Authority's Confidentiality Advisory Group (reference: 303623). Ethics approval for the LOCOMOTION study was obtained from the Bradford and Leeds Research Ethics Committee on behalf of Health Research Authority and Health and Care Research Wales (reference: 21/YH/0276). The iCARE Secure Data Environment was given favourable ethics approval by the South West—Central Bristol Research Ethics Committee (reference 21/SW/0120; IRAS project ID 282093) and includes the Whole System Integrated Care (WSIC) database (West Midlands Solihull Research Ethics Committee [reference 18/WM/0323; IRAS project ID 252449]). All data used in this paper were fully anonymised before analysis.

## Conflicts of Interest

The authors declare no conflicts of interest.

## Supporting information


**Table S1:** Demographic characteristics of the included patient population with a recorded diagnosis of Long Covid. **Table S2:** Outpatient clinic continuity of care in the post‐diagnosis period according to patient demographic characteristics.

## Data Availability

Data used in this study were held within the WSIC database hosted in the iCARE Secure Data Environment, and patient data are not available for sharing on account of privacy regulations. Code used for the study and aggregate data outputs are available from the authors upon reasonable request.
