# Supplementary material for: Exploring the Intensity and Continuity of Hospital Care for Patients With Long Covid: Evidence From an English Urban Healthcare System
Source: Health Expect. 2026 Feb 11;29(1):e70527. doi: 10.1111/hex.70527 (PMC12892402; doi:10.1111/hex.70527)
Supplement: Supplementary file 1 — Table S1: Demographic characteristics of the included patient population with a recorded diagnosis of Long Covid. Table S2: Outpatient clinic continuity of care in the post‐diagnosis period according to patient demographic characteristics. [file HEX-29-e70527-s001.docx]

Supplementary results

**Table S1:** Demographic characteristics of the included patient population with a recorded diagnosis of Long Covid.

| Variable | | N | % |
| --- | --- | --- | --- |
| Age | 18-30 | 744 | 13.4 |
|  | 31-40 | 1092 | 19.6 |
|  | 41-50 | 1233 | 22.2 |
|  | 51-60 | 1181 | 21.2 |
|  | 61-70 | 767 | 13.8 |
|  | 71-80 | 356 | 6.4 |
|  | 81-90 | 152 | 2.7 |
|  | >90 | 36 | 0.6 |
| IMD Decile (national) | 1 (most deprived) | 122 | 2.2 |
|  | 2 | 557 | 10.0 |
|  | 3 | 812 | 14.6 |
|  | 4 | 768 | 13.8 |
|  | 5 | 785 | 14.1 |
|  | 6 | 684 | 12.3 |
|  | 7 | 511 | 9.2 |
|  | 8 | 468 | 8.4 |
|  | 9 | 380 | 6.8 |
|  | 10 (least deprived) | 192 | 3.5 |
|  | Missing | 282 | 5.1 |
| Gender | Female | 3682 | 66.2 |
|  | Male | 1879 | 33.8 |
| Ethnicity | Asian or Asian British | 1690 | 30.4 |
|  | Black or Black British | 527 | 9.5 |
|  | Mixed | 229 | 4.1 |
|  | Other ethnic groups | 499 | 9.0 |
|  | White | 2599 | 46.7 |
|  | Missing | 17 | 0.3 |
| Total |  | 5561 |  |

**Table S2:** Outpatient clinic continuity of care in the post-diagnosis period according to patient demographic characteristics. Values presented are medians, with the 25th and 75th centiles in parentheses. P-values from Kruskal-Wallis tests for each continuity measure and each demographic characteristic are also shown (in bold where p▒<▒0.05).

| **Variable** | **Category** | **Number of attendances** | **Count** | | **SeCon** | |
| --- | --- | --- | --- | --- | --- | --- |
|  |  |  | **Provider** | **Specialty** | **Provider** | **Specialty** |
| **Age category** | <= 30 | 3 (1-7) | 1 (1-2) | 2 (1-3) | 0.89 (0.50-1.00) | 0.50 (0.25-1.00) |
|  | 31-40 | 4 (2-10) | 1 (1-2) | 2 (1-4) | 0.95 (0.59-1.00) | 0.47 (0.21-0.73) |
|  | 41-50 | 5 (2-10) | 1 (1-2) | 2 (1-4) | 0.89 (0.59-1.00) | 0.42 (0.19-0.75) |
|  | 51-60 | 7 (3-13) | 2 (1-2) | 3 (2-5) | 0.82 (0.57-1.00) | 0.37 (0.20-0.65) |
|  | 61-70 | 7 (3-14) | 2 (1-2) | 3 (2-5) | 0.79 (0.56-1.00) | 0.40 (0.22-0.67) |
|  | 71-80 | 8.5 (4-18) | 2 (1-3) | 4 (2-6) | 0.75 (0.52-1.00) | 0.33 (0.20-0.57) |
|  | 81-90 | 7 (2.5-14.5) | 2 (1-2.5) | 3 (1-5) | 0.73 (0.50-1.00) | 0.30 (0.13-0.50) |
|  | > 90 | 7.5 (3-16.25) | 2 (2-3) | 3 (2-5) | 0.54 (0.45-0.73) | 0.36 (0.22-0.42) |
|  | p-value | **< 0.001** | **< 0.001** | **< 0.001** | **< 0.001** | **< 0.001** |
| **IMD quintile** | 1 (most deprived) | 6 (3-12) | 2 (1-2) | 3 (1-4) | 0.82 (0.50-1.00) | 0.4 (0.21-0.67) |
|  | 2 | 5 (2-12) | 1 (1-2) | 2 (1-4) | 0.83 (0.50-1.00) | 0.44 (0.20-0.75) |
|  | 3 | 5 (2-12) | 1 (1-2) | 2 (1-4) | 0.85 (0.57-1.00) | 0.40 (0.2-0.67) |
|  | 4 | 5 (3-12) | 1 (1-2) | 2 (1-4) | 0.84 (0.57-1.00) | 0.43 (0.21-0.69) |
|  | 5 (least deprived) | 5 (2-12) | 1 (1-2) | 2 (1-4) | 0.83 (0.56-1.00) | 0.38 (0.17-0.67) |
|  | p-value | 0.6205 | 0.9741 | 0.525 | 0.9485 | **0.0287** |
| **Gender** | Female | 6 (2-12) | 1 (1-2) | 3 (1-4) | 0.85 (0.57-1.00) | 0.40 (0.20-0.67) |
|  | Male | 5 (2-11) | 1 (1-2) | 2 (1-4) | 0.81 (0.50-1.00) | 0.20 (0.40-0.67) |
|  | p-value | 0.0632 | 0.5352 | **0.0202** | 0.073 | 0.8138 |
| **Ethnicity** | Asian or Asian British | 5 (2-12) | 1 (1-2) | 2 (1-4) | 0.86 (0.56-1.00) | 0.40 (0.22-0.73) |
|  | Black or Black British | 6 (3-12) | 1 (1-2) | 3 ( 2-5) | 0.86 (0.57-1.00) | 0.44 (0.25-0.67) |
|  | Mixed | 5 (2-11) | 1 (1-2) | 3 (1-4) | 0.84 (0.57-1.00) | 0.40 (0.20-0.67) |
|  | Other ethnic groups | 5 (2-11) | 1 (1-2) | 2 (1-4) | 0.84 (0.55-1.00) | 0.40 (0.20-0.67) |
|  | White | 5 (2-12) | 1 (1-2) | 2 (1-4) | 0.82 (0.55-1.00) | 0.40 (0.19-0.67) |
|  | p-value | 0.0856 | 0.4605 | 0.1561 | 0.1999 | 0.1397 |
